# High Cysteine Membrane Proteins (HCMPs) Are Up-Regulated During *Giardia*-Host Cell Interactions

**DOI:** 10.3389/fgene.2020.00913

**Published:** 2020-08-18

**Authors:** Dimitra Peirasmaki, Showgy Y. Ma’ayeh, Feifei Xu, Marcela Ferella, Sara Campos, Jingyi Liu, Staffan G. Svärd

**Affiliations:** ^1^Department of Cell and Molecular Biology, Uppsala University, Uppsala, Sweden; ^2^Eukaryotic Single Cell Genomics Platform, Karolinska Institute, Science for Life Laboratory (SciLifeLab), Solna, Sweden; ^3^Department of Molecular Biology, Max Planck Institute for Infection Biology, Berlin, Germany; ^4^Science for Life Laboratory (SciLifeLab), Uppsala University, Uppsala, Sweden

**Keywords:** diarrhea, chromatin, RNAseq, small intestine, protozoa

## Abstract

*Giardia intestinalis* colonizes the upper small intestine of humans and animals, causing the diarrheal disease giardiasis. This unicellular eukaryotic parasite is not invasive but it attaches to the surface of small intestinal epithelial cells (IECs), disrupting the epithelial barrier. Here, we used an *in vitro* model of the parasite’s interaction with host IECs (differentiated Caco-2 cells) and RNA sequencing (RNAseq) to identify differentially expressed genes (DEGs) in *Giardia*, which might relate to the establishment of infection and disease induction. *Giardia* trophozoites interacted with differentiated Caco-2 cells for 1.5, 3, and 4.5 h and at each time point, 61, 89, and 148 parasite genes were up-regulated more than twofold, whereas 209, 265, and 313 parasite genes were down-regulated more than twofold. The most abundant DEGs encode hypothetical proteins and members of the High Cysteine Membrane Protein (HCMP) family. Among the up-regulated genes we also observed proteins associated with proteolysis, cellular redox balance, as well as lipid and nucleic acid metabolic pathways. In contrast, genes encoding kinases, regulators of the cell cycle and arginine metabolism and cytoskeletal proteins were down-regulated. Immunofluorescence imaging of selected, up-regulated HCMPs, using C-terminal HA-tagging, showed localization to the plasma membrane and peripheral vesicles (PVs). The expression of the HCMPs was affected by histone acetylation and free iron-levels. In fact, the latter was shown to regulate the expression of many putative giardial virulence factors in subsequent RNAseq experiments. We suggest that the plasma membrane localized and differentially expressed HCMPs play important roles during *Giardia*-host cell interactions.

## Introduction

Diarrheal disease is still a major cause of death in children under 5 years of age, although it is both preventable and treatable (Kotloff et al., 2013). It is also the leading cause of malnutrition in young children (Kotloff et al., 2013). *Giardia intestinalis*, also known as *Giardia lamblia* or *Giardia duodenalis*, is a unicellular protozoan parasite that infects humans and other mammals, causing the diarrheal disease giardiasis (Ankarklev et al., 2010). Giardiasis is a major cause of diarrhea worldwide with around 180 million symptomatic infections reported in humans every year (reviewed in Ryan et al., 2019). In developing countries, point prevalences range between 1.5 and 73.4%, reflecting a large number of asymptomatic infections (Farthing, 1997; Cacciò and Sprong, 2011). Elderly, malnourished or immunodeficient people are at risk of acquiring giardiasis but children are the most affected population by the disease, with reported effects on their growth, nutrition and cognitive function (Simsek et al., 2004; Prado et al., 2005; Nematian et al., 2008). The symptoms of acute giardiasis include watery diarrhea, abdominal pain, vomiting and weight loss. If the disease becomes chronic, symptoms of malabsorption (e.g., malaise and weight loss) become more prominent (Farthing, 1997; Ortega and Adam, 1997; Gascón, 2006) and children can become stunted (Rogawski et al., 2017).

Little is known about how *Giardia* causes disease; it is not invasive, does not secrete any known toxins and it causes little inflammation in the intestine (Buret, 2007, 2008; Ankarklev et al., 2010). Nevertheless, *Giardia* trophozoites can damage the intestinal epithelial cells (IECs) and the damage results in a decrease of the total absorptive area in the intestine and villus atrophy, leading to the malabsorption of water, glucose and electrolytes and maldigestion due to the loss of digestive enzymes on the IEC brush border (Scott et al., 2000; Troeger et al., 2007; Cotton et al., 2011; Humen et al., 2011; Solaymani-Mohammadi and Singer, 2011; Bénéré et al., 2012). Several reports have shown that the structural damage of the IECs is induced by *Giardia* trophozoite attachment (Magne et al., 1991; Céu Sousa et al., 2001) and release of excretory-secretory products (ESPs) like metabolic enzymes (Ringqvist et al., 2008), cysteine proteases (CPs, Liu et al., 2018), high cysteine membrane proteins (HCMPs), tenascins (Dubourg et al., 2018) and variable surface proteins (VSPs) (Ringqvist et al., 2011; Emery et al., 2016; Ma’ayeh et al., 2017; Dubourg et al., 2018).

Simple host–parasite interaction models have been established using axenic *Giardia* trophozoites from different assemblages [WB, P1 and NF (assemblage A) and GS (assemblage B)] and different intestinal cell-lines (Caco-2, HT-29, and IEC6, Roxström-Lindquist et al., 2005; Ringqvist et al., 2011; Ma’ayeh and Brook-Carter, 2012; Ferella et al., 2014). This has identified differentially expressed genes (DGEs) in both cell types and generated information about putative virulence factors (Einarsson et al., 2016a). However, these analyses have suffered from poor sensitivity of the methods used for DGE analyses, fragmented and incompletely annotated parasite genomes and problematic medium effects (Emery-Corbin et al., 2020; Jex et al., 2020).

In this study, we used our well-established model system of *Giardia* trophozoites (isolate WB) and human IECs (differentiated Caco-2 cells), combined with medium pre-incubation and medium controls, a new version of the *Giardia* WB genome (Xu et al., 2020) and an in-house RNA sequencing (RNAseq) pipeline to generate a more complete differentially expressed genes (DGE) data set from trophozoites interacting with IECs. We used the new data set together with earlier data to pin down the most commonly differentially expressed *Giardia* trophozoite genes during interactions with human IECs *in vitro*. The combined data suggest that the stress-regulated HCMPs and other cysteine-rich proteins are important during host–parasite interactions. Follow-up studies showed that up-regulated HCMPs localize to the trophozoite plasma membrane and that they are regulated by histone acetylation and levels of free-iron in the medium.

## Materials and Methods

### Cell Culture

The human colon adenocarcinoma cell line (Caco-2, clone TC7) (Roxström-Lindquist et al., 2005) was used in the experiments at a differentiated state (Lievin-Le Moal, 2013). Caco-2 cells (Passage no. 6–8) were cultured in 25 cm^2^ tissue culture flasks (T25) filled with 10 ml of complete Dulbecco’s Modified Eagle Medium (DMEM) containing 10% fetal bovine serum (FBS), 2 mM GlutaMAX (Gibco, Thermo Fisher Scientific, MA, United States), 1× MEM non-essential amino acid solution (Sigma-Aldrich, MO, United States) and 1× of penicillin-streptomycin 100× solution (10,000 units penicillin and 10 mg streptomycin/ml) (Sigma-Aldrich, MO, United States). Culture flasks were incubated in a humidified incubator (10% CO_2_ and 37°C) for 21 days until differentiation during which the media were changed twice weekly. For host–parasite interactions, heat inactivated (HI)-FBS (Gibco, Thermo Fisher Scientific) was used in the DMEM to avoid adverse effects of active serum components on trophozoites.

*Giardia intestinalis* isolate WB, clone C6 (ATCC 30957) trophozoites were cultured in 50 or 10 ml tubes filled with TYDK medium (Keister, 1983), supplemented with 10% heat-inactivated bovine serum (Gibco, Thermo Fisher MA, United States). All tubes were incubated at 37°C until reaching peak density (80% confluence) upon which they were used in the experiments. All materials used in the TYDK medium were purchased from Sigma-Aldrich unless otherwise stated.

### Cell–Cell Interactions

At the beginning of the experiment, both trophozoites and human cell cultures (T25) were washed twice with warm PBS (37°C) and replenished with fresh DMEM with 10% HI-FBS (50 ml for trophozoite culture and 9 ml for human cell culture). Incubating the trophozoites in complete DMEM prior to the experiment was deemed necessary to reduce shifts in gene transcription due to the change of media (i.e., from TYDK to DMEM). Initially, all cultures were incubated in a humidified tissue culture incubator for 2 h at 37°C after which trophozoites were processed for addition to the differentiated Caco-2 cells. This involved incubating culture tubes on ice (10 min) for detachment, counting (Neubauer Chamber slide), pelleting (centrifugation; 7 min, 750 × *g* and 4°C), resuspension in 1 ml of DMEM (3 × 10^7^ cells) and finally addition to the differentiated Caco-2 cells (T25 flasks). Trophozoites were incubated with the differentiated Caco-2 cells for 1.5, 3, and 4.5 h (10% CO_2_ at 37°C). At the end of each time point, the interaction medium was removed from the flask and the co-culture was lysed directly in 1.5 ml of lysis buffer for RNA extraction. The lysis buffer was included in the PureLink RNA Mini Kit (Ambion, Thermo Fisher Scientific) together with beta-mercaptoethanol, added directly prior to cell lysis. For the starting control, trophozoites, pre-incubated for 2 h in DMEM as above, were pelleted and lysed directly in the RNA lysis buffer. All samples were collected in RNase-free Eppendorf tubes and frozen immediately in dry ice. Samples were kept at −80°C until RNA extraction. The experiment was repeated 3 times.

### Iron Regulation of HCMPs

To minimize the heterogeneity of expressed VSPs and HCMPs in the culture, we started off by creating a clonal trophozoite population from the original WB-C6 clone using serial dilution. Briefly, a trophozoite culture was diluted in TYDK and seeded as single cells into the wells of a 96-well plate. Upon reaching 70–80% confluence, a clonal culture was selected used to seed 10 ml tubes containing TYDK, TYDK without added ferric (i.e., ferric ammonium citrate), and TYDK without ferric and supplemented with 50 μM of the metal ion chelator 2,2′-Bipyridyl (Sigma-Aldrich, MO, United States). The concentration of 2,2′-Bipyridyl was the highest that did not affect growth of *Giardia* trophozoites in TYDK. Iron analyses at ALS (Umeå, Sweden) showed that the standard TYDK contained 5.5 mg/l iron, TYDK without added iron and with added chelator 1.6 mg/ml. It should be noted that the chelator reduces the available, free iron but the total level is not decreased. Upon reaching 80% confluence, trophozoites were detached on ice (10 min), collected by centrifugation (7 min, 750 × *g*, and 4°C), washed with ice-cold PBS, pelleted (7 min, 750 × *g*, and 4°C), lysed in 1.5 ml of Trizol and collected in Eppendorf tubes. All collected samples were frozen immediately in dry ice and kept at −80°C until RNA extraction. The experiment was repeated 3 times.

### RNA Extraction, Library Preparation, and RNA Sequencing

The samples collected above were processed according to the instructions provided in the PureLink RNA Mini Kit (Ambion, Thermo Fisher scientific). A DNaseI treatment step (PureLink DNase Set, Ambion, Thermo Fisher Scientific) was performed during RNA extraction to remove genomic DNA before eluting the RNA. RNA quality was assessed by evaluating the 260/280 and 260/230 ratios (NanoDrop 1000 Spectrometer, Thermo Fisher Scientific) and electrophoresing the samples (500 ng) on a 1.5% Tris-Borate-EDTA (TBE) agarose gel containing 20 mM of guanidium isothiocyanate (GITC). Sequencing libraries were prepared from 500 ng of total RNA using the TruSeq stranded mRNA library preparation kit (Cat no. RS-122-2101/2102, Illumina Inc.), which included a polyA selection step. Libraries preparation was performed following the manufacturers’ protocol (no. 15031047). The quality of prepared libraries was evaluated using a Fragment Analyzer from Advanced Analytical (AATI) using the DNF-910 kit and they were quantified by qPCR using the Library quantification kit for Illumina (KAPA Biosystems) on a CFX384 Touch instrument (Bio-Rad) prior to cluster generation and sequencing. Sequencing was carried out on an Illumina NovaSeq6000 instrument (NVCS v 1.3.0/RTA v3.3.3) according to the manufacturer’s instructions. De-multiplexing and conversion to FASTQ format was performed using the bcl2fastq2 (2.20.0.422) software, provided by Illumina^1^. Additional statistics on sequencing quality were compiled with an in-house script from the FASTQ-files, RTA and BCL2FASTQ2 output files. RNA sequencing was performed at the SciLifeLab NovaSeq Sequencing Platform, Uppsala University, Sweden.

### Bioinformatics Analyses of RNA Sequencing Data

We have produced our own pipeline for RNAseq analyses in *Giardia* and all scripts used in the bioinformatics analyses are available upon request. Essentially STAR v020201 (Dobin et al., 2013) was used to map the RNA-Seq reads to the new, more complete *Giardia* WB reference genome (Xu et al., 2020) and the raw counts per gene were generated with the parameter “–quantMode GeneCounts.” Downstream gene differential expression analysis was carried out in R using edgeR v3.6.8 (Robinson et al., 2010) workflow. Quasi-likelihood (QL) *F*-test (glmQLFTest) was used to determine significant differential gene expression. Genes with adjusted *p*-value ≤ 0.05 were considered significant and reported here. Raw reads and the processed raw counts per gene were deposited at gene Expression Omnibus (GEO), available as accession ID GSE144004 for the interaction experiment and GSE136820 for the iron depletion experiment.

### Construction of Plasmids With Epitope-Tagged HCMPs and Transfection of Trophozoites

Three HCMPs were selected for further immunofluorescence studies (ORFs 7715, 91707, and 115066). The selection was based on their differential gene expression in all earlier published *Giardia*-host cell interaction reports (Ringqvist et al., 2011; Ma’ayeh and Brook-Carter, 2012; Ferella et al., 2014). The selected genes were PCR-amplified from genomic DNA of the WB isolate. For C-terminal HA-tagging primers were designed to amplify the gene of interest, including 100 bp upstream of the start codon (i.e., putative promoter region) and the complete codon region, except the stop codon. Adaptor sequences containing unique restriction sites were added to the 5′ end of primers and the whole sequence was checked for inframe translation. All primers were analyzed using the online tool OligoAnalyzer 3.1 – Integrated DNA Technologies (IDT) and are shown in Supplementary Table S1. Each PCR reaction contained 20 ng of DNA, 0.6 μL of 10 mM dNTPs, 1.2 μL of each forward and reverse primers (200 nM), 6 μL of 5× High Fidelity PCR buffer with MgCl_2_ (Thermo Fisher Scientific), 0.5 μL of Phusion Hot Start II High-Fidelity DNA Polymerase (proofreading – Thermo Fisher Scientific) and up to 30 μL of sterile water. PCR amplification was performed following the standard protocols, including initial denaturation and enzyme activation (98°C, 5 min), denaturation (15 s), annealing (variable annealing temperature based on primers, see Supplementary Table S1) and primer extension (35 cycles, 72°C for 80 s), and a final extension step at 72°C for 5 min. Amplicons were gel-purified using the GeneJET Gel Extraction Kit (Thermo Fisher Scientific) according to the manufacturer’s instructions. The purified products were double-digested with the appropriate restriction enzymes (HindIII and EcoRI for ORFs 7715 and 91707 and XbaI and EcoRI for ORF 115066) and ligated into the episomal pPAC-3xHA-C-terminus plasmid vector cleaved with the same restriction enzymes as previously described (Jerlström-Hultqvist et al., 2012). Plasmids (∼20 μg) were transfected into *Giardia* trophozoites (Jerlström-Hultqvist et al., 2012) and the transfectants were grown at 37°C under puromycin selection (50 μg/ml) to establish a stable transfectant line.

The same plasmids were used to produce integrated C-terminal constructs. The C-terminal constructs were cleaved with restriction enzymes with unique sites in each HCMP gene (7715-Sph1, 91707-MfeI, and 115066-PmaC1), generating linearized constructs that were gel purified and 20 μg linearized plasmid was used in transfection as above. Genomic PCRs using primers outside of the construct and in the plasmids were used to verify that the plasmids were integrated into the correct genomic sites.

The three HCMPs were also cloned with N-terminal HA tags downstream of the signal sequence of each HCMP, followed by the complete HCMP gene sequences, including the stop codons. The cloning was done in two steps, generating two PCR products that were fused and cloned into the pPAC plasmid without the HA tag (cut at the EcoRI and SmaI site for ORFs 7715 and 91707 and XbaI and SmaI sites for ORF 115066). Plasmids were transfected into *Giardia* trophozoites as previously described and positive clones were selected under puromycin pressure (50 μg/ml).

### Immunofluorescence of HCMP Transfectants

Transfectants were examined by immunofluorescence for the localization of the HCMPs. Fifteen microliter of trophozoite culture were pipetted onto a poly-L-lysine coated microscopy slide and allowed to attach for 5 min at 37°C in a humidified chamber. Attached trophozoites were fixed for 20 min using 15 μl of 4% paraformaldehyde (PFA) in PBS and incubated at 37°C. The fixative was then removed using vacuum suction and neutralized with 15 μl of 0.1 M glycine dissolved in PBS. Cells were rinsed in PBS and permeabilized with 0.1% Triton-X 100 in PBS for 30 min at 37°C, washed with PBS and blocked overnight (4°C) in a blocking solution (2% bovine serum albumin dissolved in PBS and 0.05% Triton-X 100). Next day, the blocking solution was removed and each well of the slide was incubated with 20 μl of an Anti–HA mouse monoclonal antibody (1:500 dilution, Sigma-Aldrich) for 1 h at room temperature. Next, the antibody was removed using vacuum suction and the cells were washed with PBS followed by 1-h incubation at room temperature with 20 μl of Alexa Fluor 488-conjugated Goat anti-mouse antibody (1:250 dilution, Thermo Fisher). Finally, antibodies were removed and the wells were washed with PBS and mounted using Vectashield containing the DNA stain 4′, 6′-diamidino-2-phenyldone (DAPI) (Vector Laboratories, place and country). The slides were stored at −20°C in darkness. The slides were examined using a Zeiss Axioplan2 fluorescence microscope and the images were processed using the software Axiovision Rel. 4.8.

Transfectants were also followed by IFA for 37 generations after initial transfection. Two hundred cells were counted each time and valuated according to the cellular localization of the expressed protein.

### Oxidative Stress Regulation of HCMPs

To study the medium effects on HCMP expression we made use of our C-terminally HA-tagged episomal constructs of HCMP 7715, 91707, and 115066. Transfected parasites (1 × 10^7^ cells) were incubated for 3 h in 6-well plates filled with medium at 5 different conditions: TYDK, DMEM, DMEM + Caco-2 cells, DMEM + 1 mM cysteine, and DMEM + 1 mM cysteine + Caco-2 cells. Trophozoites were detached on ice (10 min), collected by centrifugation (7 min, 750 × *g*, and 4°C), washed with ice-cold PBS, pelleted (7 min, 750 × *g* and 4°C), lysed in 1.5 ml of Trizol and collected in Eppendorf tubes. All collected samples were frozen immediately in dry ice and kept at −80°C until RNA extraction. The experiment was repeated 3 times.

### Chromatin Regulation of HCMPs

To determine whether the HCMPs are epigenetically regulated, we used inhibitors of histone deacetylases (HDACs) to examine their effect on RNA levels of selected HCMPs showing differential gene expression in this and earlier gene expression studies; GL50803_7715, GL50803_9620, GL50803_11309, GL50803_91707, and GL50803_115066. The inhibitors of NAD^+^-independent HDAC inhibitors [trichostatin A (TSA) and sodium butyrate (NaB)] and the inhibitor of NAD^+^-dependent HDACs [nicotinamide (Nt)] have been previously shown to interfere with epigenetic regulation in *Giardia* (Carranza et al., 2016) and thus, they were selected for this study. The earlier reported conditions for treatment were used and trophozoites (10^4^ starting trophozoites) were incubated in TYDK for 1 or 3 days with each inhibitor at a final concentrations of 200 nM for TSA, 20 nM for NaB and 10 nM for Nt (Carranza et al., 2016). At the end of all incubations, treated trophozoites were detached from the tubes wall (10 min on ice), pelleted (750 × *g*, 4°C and 10 min) and lysed in 1.5 ml of Trizol (Ambion, Thermo Fisher Scientific) and transferred into Eppendorf tubes. For the control, trophozoites incubated for the same duration without the inhibitor were processed as stated previously and lysed directly in Trizol. All collected samples were frozen immediately in dry ice and kept at −80°C until RNA extraction. RNA extraction was performed according to the instructions provided with the Trizol reagent. Extracted RNA was checked for quality as stated in a previous section. The experiment was repeated four times.

### Quantitative Polymerase Chain Reaction (qPCR)

High quality intact RNA samples were processed for cDNA synthesis. First, 1 μg of each sample was DNase I (Fermentas, Thermo Fisher) treated and the DNA-free RNA was reverse transcribed according to the instructions in the Revert Aid H Minus cDNA Synthesis Kit (Thermo Fisher Scientific). Oligo-(dT)_18_ primers were used for cDNA synthesis. cDNA reaction mixtures were diluted (1.5–5 ng template per reaction) and used in the qPCR reactions together with 250 nM of each primer and the Maxima SYBR Green qPCR Master Mixes (Thermo Fisher Scientific). Reactions were set up in 20 μl volume and run following the manufacturer’s instructions with the inclusion of melt curve analysis in the end of the run. qPCRs were performed in a StepOnePlus thermal cycler (Applied Biosystems, Carlsbad, CA, United States). All primers (Sigma-Aldrich) used in the qPCRs are listed in Supplementary Table S1. The gene encoding tryptophanyl-tRNA-synthetase (TtRNA) (GL50803_3032) was used as an endogenous control in the qPCR reactions (Einarsson et al., 2016b). The fold change in gene expression was calculated using the ΔΔ*C*t method. Significant changes in RNA levels between treatments and controls were assessed using one-way analysis of variance (ANOVA) at α < 0.05 followed by the Dunnett’s multiple comparison test at *P* < 0.05.

## Results

### RNAseq Analyses of *Giardia*-Host Cell Interactions

**FIGURE 1:**
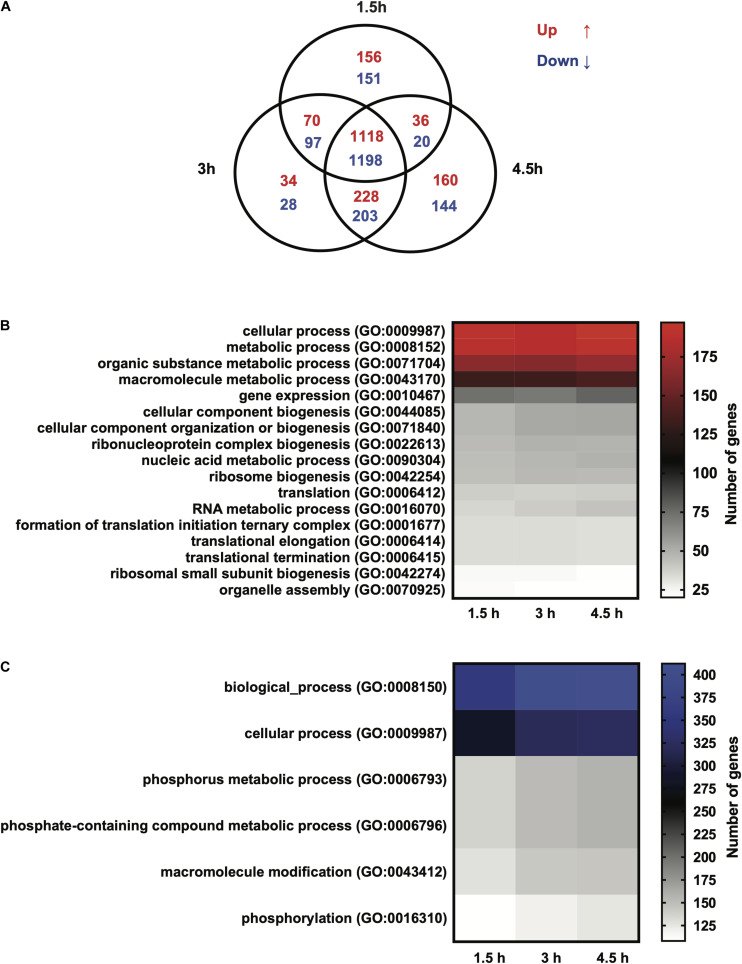
**(A)** Venn diagram showing the number of differentially expressed genes (DEGs) in *Giardia intestinalis* WB trophozoites incubated with differentiated Caco-2 cells *in vitro* for 1.5, 3, and 4.5 h. **(B)** Gene Ontology (GO) analysis of the DEGs at the three time points represented by a heat map. The map shows the up-regulated DEGs enriched for biological functions. **(C)** Gene Ontology (GO) analysis of DEGs at the three time points represented by a heat map. The map shows the down-regulated DEGs enriched for biological functions. Note that up-regulated (red) and down-regulated (blue) heat maps are color-coded.

RNA sequencing was performed on *Giardia intestinalis* trophozoites (isolate WB) incubated with and without differentiated Caco-2 cells, in three biological replicates. A major difference in this experiment compared to earlier studies was a 2 h pre-incubation of the trophozoites in DMEM before addition to the Caco-2 cells in order to reduce the medium effects that have been seen to dominate gene expression changes in earlier experiments (Ringqvist et al., 2011; Ma’ayeh and Brook-Carter, 2012; Ferella et al., 2014). We also used the new version of the *Giardia* WB genome, which is more complete and better annotated (Xu et al., 2020). EdgeR was used to analyze the RNA-Seq reads to obtain the differentially expressed genes (DEGs). Overall, an average of 40.4 million paired-end reads were obtained for the starting trophozoite population (DMEM pre-incubated trophozoites), amongst which 79.4% mapped to the *Giardia* WB reference genome (Xu et al., 2020). For trophozoites co-incubated with IECs, we obtained 42.3, 40.4, and 44.7 million paired-end reads on average at the 1.5, 3, and 4.5 h time points, with 53.1, 51.3, and 47.4% of the respective reads mapped to the *Giardia* WB genome. We did comparisons of gene expression between starting trophozoites and trophozoites after different times of interaction with Caco-2 cells. EdgeR identified 2,840, 2,968, and 3,103 DEGs (adjusted *p*-value ≤ 0.05) for each timepoint compared to the starting trophozoites, and 2,306 of the DEGs were shared between all timepoints (Figure 1A). The overlap of DEGs between the different timepoints increases with time and there are less unique DEGs at 3 h (Figure 1A). Comparisons between each time-point generated only 2DEGs from the 1.5–3 h comparison (HCMP114089 and FixW6289) and none from the 3–4.5 h comparison. Detailed information about all the DEGs can be found in Supplementary Table S2 (1.5 h), Supplementary Table S3 (3 h), and Supplementary Table S4 (4.5 h). For each time point, 61, 89, and 148 DEGs were up-regulated more than twofold (log_2_FC > 1) whereas 209, 265, and 316 DEGs were down-regulated more than twofold (log_2_FC < −1), respectively (Supplementary Tables S2–S4). A total of 35 genes were up-regulated threefold or more (Table 1). Only five of these DEGs were found among the 30 most up-regulated genes in a microarray-based study of trophozoites co-incubated with differentiated Caco-2 cells (ORFs 5800, 10659, 114210, 115066, and 137727) (Ringqvist et al., 2011), showing the effects of the different approaches. It was also a partial overlap with genes up-regulated in three different *Giardia* isolates (WB, P-1, and NF) incubated with murine IEC6 cells (ORFs 5800, 114210, 115066, and 137727) (Ma’ayeh and Brook-Carter, 2012). Three of the commonly up-regulated DEGs (ORFs 10659, 115066, and 137727) are HCMPs and it is the most represented gene family (8 of 35 genes, 23%) in Table 1. The majority of the 32 DEGs down-regulated fourfold or more were down-regulated already at 1.5 h (Table 2). Several of these DEGs are involved in the regulation of cell cycle such as MAD2 (ORF100955) and cyclin (ORF 17400) as well arginine metabolizing enzymes (e.g., arginine deiminase, and carbamate kinase), suggesting a reduced arginine consumption and replication of the parasites already after 1.5 h co-incubation.

**TABLE 1:** Genes up-regulated more than threefold during co-incubation of *Giardia intestinalis* WB isolate trophozoites with differentiated Caco-2 cells *in vitro* for 1.5, 3, and 4.5 h.

**Gene ID**	**Description**	**1.5 h**	**3 h**	**4.5 h**
GL50803_3470	ABC transporter family protein	4.6	6.1	6.1
GL50803_137727	High cysteine membrane protein EGF-like	4.0	4.3	4.6
GL50803_5800	Lipid binding protein	3.2	4.0	4.6
GL50803_26679	Hypothetical protein	2.1	3.7	4.3
GL50803_d15250	High cysteine membrane protein Group 6	3.2	4.3	4.0
GL50803_8227	ATP-binding cassette protein 5	3.5	4.3	3.7
GL50803_27713	Hypothetical protein	1.4	2.3	4.0
GL50803_102110	Chromosome segregation protein SMC	NA	2.6	4.0
GL50803_17121	Bip	4.0	2.1	2.0
GL50803_114089	High cysteine membrane protein Group 4	NA	2.3	3.7
GL50803_8883	Hypothetical protein	2.0	3.0	3.7
GL50803_114210	Hypothetical protein	1.6	2.5	3.7
GL50803_7195	Glutamate synthase	3.5	3.7	3.5
GL50803_115066	High cysteine membrane protein VSP-like	2.5	3.0	3.5
GL50803_32419	Hypothetical protein	2.3	3.2	3.5
GL50803_113415	Hypothetical protein	2.6	3.0	3.5
GL50803_16568	Transcription factor, putative	3.5	3.0	3.5
GL50803_16424	Disk-associated protein	2.0	2.5	3.2
GL50803_9620	High cysteine membrane protein Group 2	2.3	3.2	3.2
GL50803_11309	High cysteine membrane protein Group 1	NA	2.5	3.2
GL50803_4191	Kinase, CMGC CDK	NA	1.6	3.1
GL50803_61026	Hypothetical protein	2.2	2.9	3.1
GL50803_14164	Ankyrin repeat protein 1	2.1	2.3	3.0
GL50803_10659	High cysteine membrane protein Group 1	1.9	2.5	3.0
GL50803_8377	Putative TM nitroreductase	2.1	2.5	3.0
GL50803_112885	Adenine phosphoribosyltransferase	1.9	2.5	3.0
GL50803_22547	High cysteine membrane protein Group 2	2.6	2.8	3.0
GL50803_26835	PIG-X/PBN1 family protein	1.9	2.5	3.0
GL50803_22504	Hypothetical protein	2.3	2.6	3.0
GL50803_34093	Ribosomal L38e	2.0	2.3	3.0
GL50803_14670	Protein disulfide isomerase PDI3	3.0	2.1	2.3
GL50803_114636	Hypothetical protein	2.6	3.0	2.8
GL50803_15450	Hypothetical protein	2.8	3.0	3.0
GL50803_29692	Hypothetical protein	NA	2.7	3.0

**Expression values in fold change*.*

**TABLE 2:** Genes down-regulated more than fourfold during co-incubation of *Giardia intestinalis* WB isolate trophozoites with differentiated Caco-2 cells *in vitro* for 1.5, 3, and 4.5 h.

**Gene ID**	**Description**	**1.5 h**	**3 h**	**4.5 h**
GL50803_21116	Kinase, CMGC CMGC-GL1	0.1	0.1	0.1
GL50803_100955	Mitotic spindle checkpoint protein MAD2	0.2	0.1	0.1
GL50803_112103	Arginine deiminase	NA	0.2	0.1
GL50803_17090	Giardia trophozoite antigen GTA-1	0.3	0.2	0.1
GL50803_87577	Hypothetical protein	0.2	0.2	0.1
GL50803_16367	Hypothetical protein	0.2	0.1	0.1
GL50803_102322	Glycosyltransferase, putative	0.2	0.1	0.1
GL50803_10527	Hypothetical protein	0.2	0.2	0.1
GL50803_17400	Cyclin	0.2	0.2	0.1
GL50803_16534	Ankyrin repeat protein 1	0.2	0.2	0.2
GL50803_7538	Hypothetical protein	0.2	0.2	0.2
GL50803_8044	Seven transmembrane protein 1	0.2	0.2	0.2
GL50803_7137	Hypothetical protein	0.2	0.2	0.2
GL50803_16802	Kinase, CMGC CDK	0.2	0.2	0.2
GL50803_8263	Hypothetical protein	0.2	0.2	0.2
GL50803_5188	Ankyrin repeat protein 1	0.3	0.2	0.2
GL50803_86933	Hypothetical protein	0.2	0.2	0.3
GL50803_11940	Hypothetical protein	0.3	0.2	0.3
GL50803_95593	Kinase, NEK	0.3	0.2	0.2
GL50803_13651	Hypothetical protein	0.3	0.2	0.2
GL50803_16453	Carbamate kinase	0.5	0.3	0.2
GL50803_14971	SMC family protein	0.3	0.2	0.2
GL50803_8726	Hypothetical protein	0.3	0.2	0.2
GL50803_8980	Hypothetical protein	0.3	0.2	0.2
GL50803_7390	tRNA-dihydrouridine synthase 2	0.4	0.2	0.2
GL50803_16343	Median body protein	0.3	0.3	0.2
GL50803_11151	Hypothetical protein	0.3	0.3	0.2
GL50803_113610	GlcNAc-PI synthesis protein	0.3	0.3	0.2
GL50803_2622	Phosphatidylinositol-4-phosphate 5-kinase	0.4	0.3	0.25
GL50803_17558	Kinase, CMGC DYRK	0.3	0.3	0.25
GL50803_13133	Hypothetical protein	0.3	0.3	0.25
GL50803_5183	Hypothetical protein	0.3	0.25	0.25

**Expression values in fold change*.*

A GO term analysis for the enrichment of biological terms within the up-regulated DEG population at the three time points showed overlapping functions, involving cellular processes, metabolic processes, gene expression, ribosome biogenesis and translation (Figure 1B). The commonality in these biological functions can be explained by the high number of overlapping DEGs among the three time points. A further curation of DEGs for the enrichment of molecular functions produced groups for catalytic activity, hydrolase activity, RNA binding, nucleic acid binding and ribosome biogenesis (Supplementary Figure S1). Exclusive to the 1.5 h time point was the emergence of the functional groups, peptidase activity (GO:0008233, 32 DEGs) and oxidoreductase activity (GO:0016491, 25 DEGs). The peptidase activity group contained genes encoding cysteine proteases (e.g., ORFs 10217, 15564, 114915, and 137680), metalloproteases (e.g., ORFs 9508, 16823, and 93551), proteasome subunits (e.g., 3209, 13127, 33166, and 91643) and HCMPs with putative leishmanolysin-like peptidase activity (ORFs, 9620, 112432, and 137715) (Supplementary Table S2). The oxidoreductase group contained genes encoding proteins with peroxidase activity including the peroxiredoxins, nitroreductases and NADH oxidase (e.g., ORFs 3042, 6289, 9355, 9719 14521, 15009, 15307, 16076, 22677, and 23888). We further analyzed the down-regulated DEGs at the three time points for GO terms and this showed an enrichment of the biological functions, cellular processes, metabolic process and phosphorylation including phosphate-containing compounds metabolism (Figure 1C). A molecular functions query, on the other hand, showed many functions associated with binding such as organic cyclic compounds, nucleotides ribonucleotide, nucleoside phosphate, ATP, carbohydrate derivatives and ions (Supplementary Figure S1). Analyses of the 3 and 4.5 h time points for GO terms produced more molecular groups with the down-regulated DEGs, including transferase activity (transferring phosphorus-containing groups), phosphotransferase activity, kinase activity and hydrolase activity (Supplementary Figure S1). Overall, the above changes show the complexity of cellular and metabolic responses in *Giardia* trophozoites incubated with IECs.

In total 53 DEGs encode HCMPs that are expressed in at least two timepoints (Supplementary Table S5). 34 of the differentially expressed HCMPs were up-regulated with 13 being up-regulated more than twofold (Supplementary Table S5). On the other hand, 19 HCMPs were down-regulated at least two timepoints with four that were down-regulated to less than 0.5-fold (Supplementary Table S5). In addition, there were 17 HCMPs that were only identified at specific interaction time points; 6 (7 up- and 3 down-regulated) at 1.5 h, 1 (down-regulated) at 3 h and 10 (5 up- and 5-down-regulated) at 4.5 h (Supplementary Table S5). Overall, these results show that 70 of a total of 116 (60%) HCMP genes in the WB genome are differentially expressed during trophozoite-IEC interactions.

### Localization of Up-Regulated HCMP Proteins During *Giardia*-Host Cell Interactions

**FIGURE 2:**
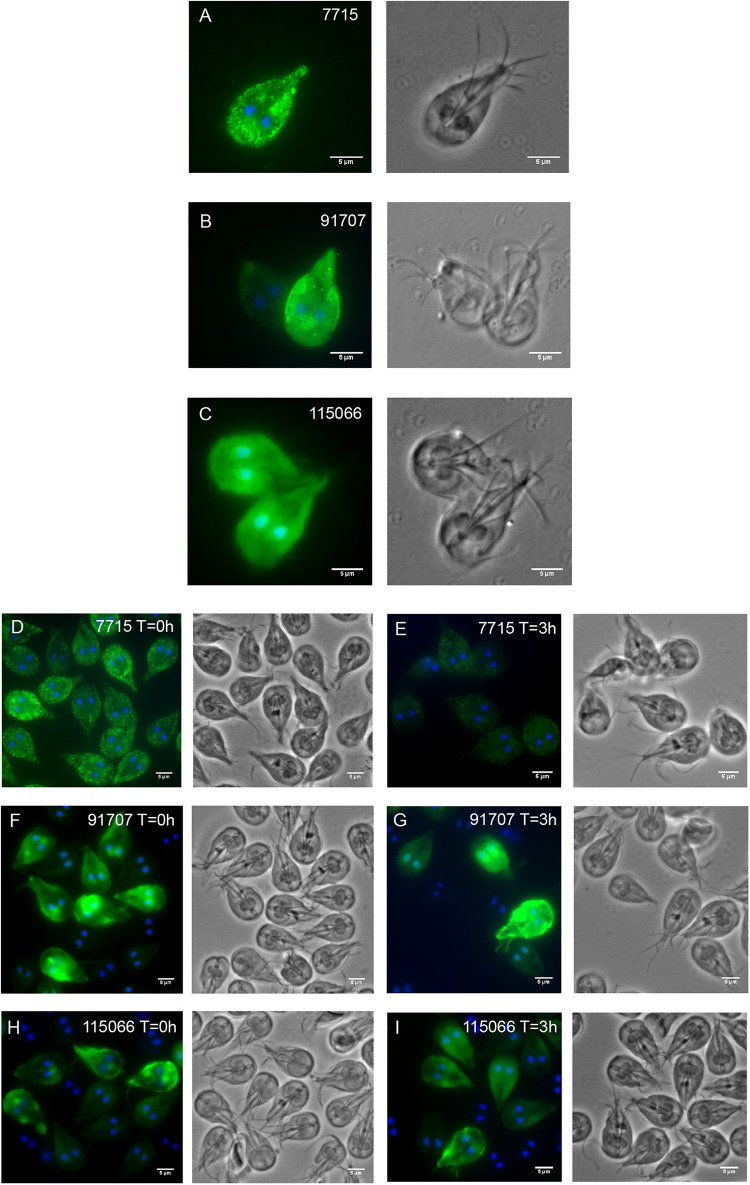
Fluorescent microscopy analyses of three C-terminally HA-tagged, episomally expressed HCMPs. Panels **(A–C)** (**A**-7715, **B**-91707, and **C**-115066) show *Giardia* trophozoites after axenic growth to 80% confluence in TYDK. The 3 transfectants were also co-incubated with human IECs **(D–I)** and epitope tagged HCMP localization was studied at the start of interaction (*T* = 0 h, **D,F,H**) and after 3 h interaction with differentiated Caco-2 cells (*T* = 3 h, **E,G,I**).

Due to the lack of expression and localization data of most HCMPs, three HCMP genes (HCMP 7715, 91707, and 115066) that were differentially expressed during IEC interactions in our and in earlier studies (Ringqvist et al., 2011; Ma’ayeh and Brook-Carter, 2012; Ferella et al., 2014) were selected for further characterization and cloned in episomal *Giardia* expression-vectors tagged with a 3XHA epitope at the C-terminus (Jerlström-Hultqvist et al., 2012). Microscopic examination of the transfected parasites revealed a plasma membrane localization for all 3 HCMPs (Figures 2A–C), combined with spotty cytoplasmic fluorescence for HCMPs 7715 and 91707 (Figures 2A,B). The initial plasma membrane localization of the recently transfected, episomal 91707-HA construct (Figure 2B) gradually changed to a more internal localization around the pheripheral vesicles (PVs) during later generations (Supplementary Figure S2), whereas the other two constructs were stable for at least 30 generations. In order to verify that the localization from the episomal constructs is not an artifact due to over-expression we also produced integrated versions of the C-terminally tagged HCMPs. The episomal vectors were cut in the coding sequences of the HCMPs and the constructs were integrated into their cognate chromosomal loci as in Gourguechon and Cande (2011). The integrated constructs showed a spotty plasma membrane localization for all three HCMPs (Supplementary Figure S3) and more than 80% of the cells were positive, compared to 50% or less in the episomal constructs (Supplementary Figure S2). However, the immunofluorescence signal per cell was weaker, suggesting lower expression. Co-staining with Cellmask, a plasma membrane stain, supported the localization to the plasma membrane of HCMP115066 (Supplementary Figure S3). For HCMPs with N-terminal HA-fusions, the expressed proteins stayed in the ER, suggesting a disturbed protein transport (Supplementary Figure S4).

To gain further insights into the localization of the selected HCMPs in co-culture with IECs, the three C-terminal episomal HA fusion transfectants were added to differentiated Caco-2 cells and the localization of the HCMPs was examined after 0 and 3 h of co-incubation (Figures 2D–I). The interactions were performed in DMEM as opposed to the localization above, which were performed after growth in *Giardia* growth medium (TYDK). All examined HCMPs localized to the plasma membrane initially (Figures 2D,F,H). After 3 h co-incubation with IECs, the signal for HCMP 7715 was lost on the parasites (Figure 2E). It is possible that this protein had been secreted since we could previously detect HCMP 7715 in the culture supernatant during co-incubation with IECs (Ma’ayeh et al., 2017). For the HCMPs 91707 and 115066, a stronger signal could be detected at the plasma membrane and within the cytoplasm (Figures 2G,I).

### Regulation of HCMP Genes During *Giardia*-Host Cell Interactions

**FIGURE 3:**
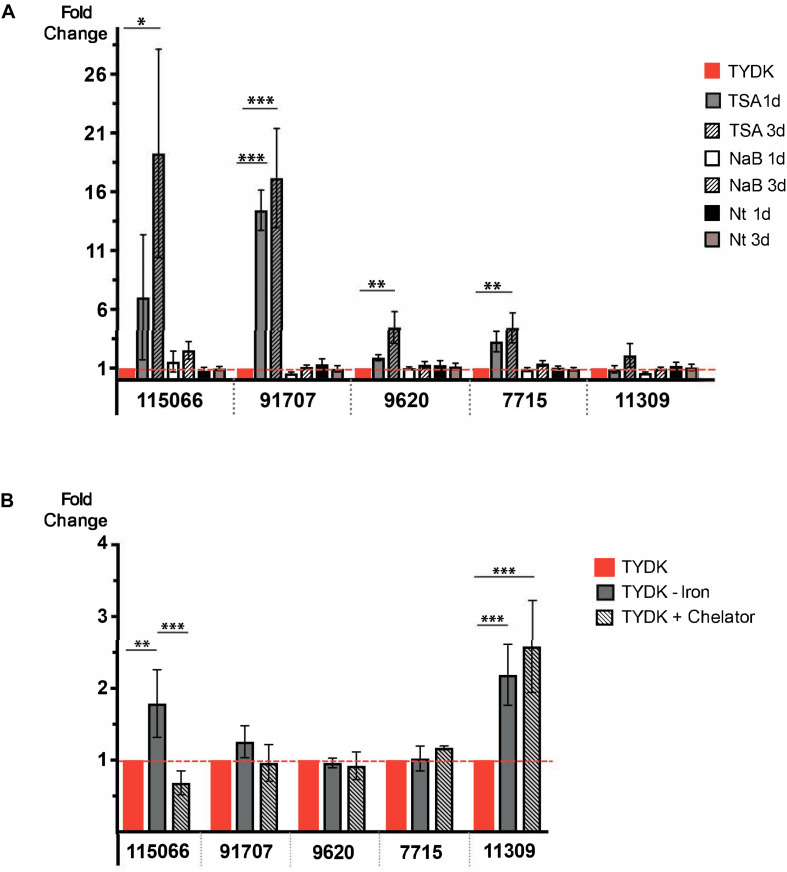
**(A)** Gene expression analyses of five HCMP genes (HCMP 9620, 7715, 11309, 91707, and 115066) during growth of trophozoites (10^4^ starting trophozoites) for 1 and 3 days in the presence of three HDAC inhibitors [Trichostatin A (TSA), Sodium butyrate (NaB) and Nicotinamide (Nt)]. The experiment was repeated four times. **(B)** Expression of HCMPs 9620, 7715, 11309, 91707, and 115066 in medium containing different levels of iron; TYDK medium with extra iron (TYDK+ Iron), TYDK medium without extra iron (TYDK– Iron) and TYDK without extra iron and addition of 50 μM of the metal ion chelator 2,2′-Bipyridyl (TYDK-Iron + Chelator). The experiment was repeated three times. Levels of expression were measured by qPCR in both panels. Significant results are indicated by ^∗^*P* < 0.05, ^∗∗^*P* < 0.01, ^∗∗∗^*P* < 0.001.

Histone deacetylases (HDACs) regulate the expression of VSPs (Carranza et al., 2016), whose gene organization and structure are similar to that of HCMPs (Xu et al., 2020). Earlier studies of the effect of an inhibitor specific for NAD^+^-independent HDACs (FR235222) on *Giardia* showed that it affected the expression of both VSPs and HCMPs (Sonda et al., 2010). We, therefore, decided to test whether the HCMPs up-regulated upon IEC contact can be epigenetically regulated using three different HDAC inhibitors that have been shown to interfere with NAD^+^-dependent (Nicotinamide, Nt) and NAD^+^-independent [Trichostatin A (TSA) and sodium Butyrate (NaB)] HDACs. Trophozoites were treated with TSA, NaB, or Nt at the same concentrations (TSA 200 nM, NaB 20 nM, and Nt 10 mM) and time (1 day) as used in earlier studies of VSP expression (Carranza et al., 2016) but with an extension to 3 days to get 80% confluent tubes and see long-term effects. Changes in the RNA levels of 5 differentially expressed HCMPs during interaction with differentiated Caco-2 cells (7715, 9620, 11309, 91707, and 115066) were studied using qPCR (Figure 3A). Neither NaB nor Nt had any effects on the RNA levels of the selected HCMPs. Nevertheless, trophozoites treated with TSA showed a significant increase in the RNA levels ranging between 4- and 16-fold for the HCMPs 7715, 9620, 91707, and 115066. Therefore, our results indicate that some of the differentially expressed HCMPS during *Giardia-*IEC interactions are regulated by NAD^+^-independent HDACs like VSPs.

Stress with metronidazole, hydrogen peroxide or growth at 39°C affect expression of certain HCMPs (Ansell et al., 2016). It is possible that oxidative stress in the DMEM medium, due to a much lower level of cysteine compared to in TYDK, is inducing the expression of the HCMPs. We tested this by growing our episomal transfectants for 3 h in different media and in the absence or presence of Caco-2 cells and the HCMP transcript levels were analyzed with qPCR (Supplementary Figure S5). It is clear that the change of medium from TYDK to DMEM has a large effect on HCMP expression but inclusion of 1 mM cysteine in the DMEM medium rather increased the HCMP expression (twofold, Supplementary Figure S5). The presence of IECs significantly increased the expression of the episomal HCMPs but inclusion of 1 mM cysteine reduced the level of up-regulation (Supplementary Figure S5). Thus, oxidative stress can affect the expression of the HCMPs but this is not the only factor leading to up-regulation during trophozoite-Caco-2 interactions.

Oxidative stress in cells is known to be associated with the cellular levels of free iron ions and DMEM contain much less free iron (0.1 mg/l) compared to TYDK (5.5 mg/l). We therefore tested whether HCMPs can be regulated by iron. To do this, we grew *Giardia* trophozoites in complete TYDK medium and in TYDK media without addition of extra iron (1.6 mg/l) with or without the addition of 50 μM of the metal ion chelator 2,2′-Bipyridyl (see section “Materials and Methods”). RNA was extracted and qPCR was used to study changes in RNA levels of the three HCMP genes. No significant differences in the RNA levels were seen for the HCMPs 91707, 9620, and 7715 in response to growth in medium with threefold less iron (Figure 3B). However, the lower levels of iron significantly increased the RNA levels of HCMP 115066 (1.7-fold) and HCMP 11309 (2.3-fold, Figure 3B). A further increase in the RNA levels of HCMP 11309 was seen when iron was chelated in the medium, whereas this reduced expression of HCMP115066 (Figure 3B). These findings suggest that iron might play a role in the regulation of HCMPs.

### *Giardia* Responses to Iron Depletion

The observed effects of iron on the expression of a small set of HCMPs and the fact the iron is a well-known regulator of virulence in intestinal bacteria (Begg, 2019) and protozoa (Gastelum-Martínez et al., 2018) inspired us to study the effects of iron on the total *Giardia* transcriptome. RNA Sequencing was performed on trophozoites grown in media with different levels of free iron to assess global changes in gene expression. We used a recently established clone of the WB-C6 isolate (see section “Materials and Methods”) in order to be able to better study the expression of variable gene families like HCMPs and VSPs. Overall, reducing the iron in the *Giardia* growth medium threefold (TYDK-Fe) resulted in 225 DEGs; 205 were up-regulated and 20 were down-regulated (Supplementary Table S6). Six genes were up-regulated twofold or more, including the HCMPs 24880 and 11309 and the iron containing enzyme pyruvate-flavodoxin oxidoreductase (PFOR, ORF17063). Among the most up-regulated genes we noted several other HCMPs (ORFs 6372, 103454, 113987, and 115066), another PFOR (ORF 114609) and several putative cation transporters (ORFs 32658, 92246, and 96670). We also detected higher expression of the secreted, putative giardial virulence factors cathepsin B 14019 and 16779 (Liu et al., 2018, 2019) and two tenascin-like proteins (ORFs 95162 and 114815) (Dubourg et al., 2018).

Chelation of free iron in the *Giardia* medium using 50 μM of 2,2′-Bipyridyl (TYDK-Fe + Chelator) resulted in 613 DEGs with 185 being up-regulated and 428 down-regulated (Supplementary Table S7). Four of the genes were up-regulated twofold or more, including HCMPs 11309 and 25816. Among the up-regulated genes we also noticed several encystation-specific genes (Cyst-wall proteins -1 and -2 and glucosamine-6-phosphate), oxidative stress related genes (Peroxoredoxin-1, superoxide reductase, Ferredoxin, Thioredoxin reductase and NADPH oxidoreductase) and histones (H2A, H3, and H4). Only two VSPs were found among the DEGs (ORFs 33279 and 111933) in TYDK-Fe whereas 15 other VSP genes were up-regulated in the presence of the chelator (Supplementary Table S7). Totally 15 HCMPs were up-regulated when iron was not added to the TYDK medium, whereas 10 of the HCMPs were differentially expressed (5 up- and 5 down-regulated) in response to chelation of metal ions (Table 3). Only 11 DEGs (9 up- and 2 down-regulated) were common between the TYDK-Fe and TYDK-Fe + chelator conditions and the HCMPs 11309, 16716, 25816, and 115066 were found to be DEGs in both treatments (Table 3). The level of chelator used in the experiments was set at the highest concentration that did not affect the growth rate of *Giardia* WB trophozoites but 2,2′-Bipyridyl also chelate other metal ions, giving broader effects than the reduction of iron in the medium. This can explain the differences between the data sets and the large number of down-regulated genes in the presence of the chelator. The effect of metal ion chelation produced an enrichment of general GO terms such as nucleic acid binding, RNA binding, transporter activity or structural constituent of the ribosome and therefore we analyzed the DEGs further using the complete biological and molecular functions. This, in turn, enriched for many biological and molecular GO terms such as translation, gene expression, ribosome biogenesis and others involving the metabolism of nucleosides, pyrimidine nucleosides, glycosyl- and organonitrogen compounds, macromolecules synthesis and amino acid activation. Cellular oxidant detoxification (GO: 0098869) also emerged within the enriched group, indicating a relation between iron and oxidative stress.

**TABLE 3:** Differentially expressed high cysteine membrane proteins (HCMPs) in *Giardia intestinali*s WB isolate trophozoites in response to different levels of iron.

**Gene ID**	**Description**	**TYDK-Fe**	**TYDK-Fe + Chelator**
GL50803_24880	High cysteine membrane protein Group 2	2.4	NA
GL50803_103454	High cysteine membrane protein Group 1	1.8	NA
GL50803_113987	High cysteine membrane protein Group 3	1.8	NA
GL50803_6372	High cysteine membrane protein	1.7	NA
GL50803_16936	High cysteine membrane protein EGF-like	1.7	NA
GL50803_15317	High cysteine membrane protein Group 1	1.6	NA
GL50803_112135	High cysteine membrane protein VSP-like	1.6	NA
GL50803_114991	High cysteine membrane protein EGF-like	1.6	NA
GL50803_103943	High cysteine membrane protein Group 3	1.5	NA
GL50803_27717	High cysteine membrane protein Group 3	1.4	NA
GL50803_21321	High cysteine membrane protein Group 5	1.3	NA
GL50803_11309	High cysteine membrane protein Group 1	2.3	2.9
GL50803_25816	High cysteine membrane protein Group 1	1.7	2.8
GL50803_115066	High cysteine membrane protein VSP-like	1.7	0.7
GL50803_16716	High cysteine membrane protein Group 5	1.3	0.8
GL50803_114617	High cysteine membrane protein Group 4	NA	1.2
GL50803_101589	High cysteine membrane protein	NA	1.2
GL50803_112432	High cysteine membrane protein Group 5	NA	1.2
GL50803_104087	High cysteine membrane protein	NA	0.8
GL50803_91707	High cysteine membrane protein Group 1	NA	0.7
GL50803_14017	High cysteine membrane protein Group 2	NA	0.6

## Discussion

Several mechanisms have been proposed to be important for induction of symptoms during a *Giardia* infection but there is yet no consensus. However, data generated during the last years have shed light on the disease mechanism, which seems to be multi-factorial (Einarsson et al., 2016a). Most of this data has been generated using different *in vitro* models of giardiasis using human or rat intestinal epithelial cell-lines combined with trophozoites or parasite extracts and it has been complemented by experimental infections in mice, gerbils and data from human giardiasis patients (Emery-Corbin et al., 2020; Jex et al., 2020). Various strategies have been developed to study the interplay and the outcome of interaction between *Giardia* and host cell *in vitro* and *in vivo* (Roxström-Lindquist et al., 2005; Ringqvist et al., 2011; Ferella et al., 2014; Kraft et al., 2017; Ma’ayeh et al., 2017). Here, we have used our well-characterized *in vitro* model of interaction between differentiated Caco-2 cells and *G. intestinalis* WB trophozoites (Roxström-Lindquist et al., 2005) combined with RNA sequencing to study transcriptional changes in trophozoites during the early hours of co-incubation with the human cells. We reasoned that early hours on interaction are important for establishing infection, counteracting intestinal epithelial cell defenses and the expression of virulence factors. One problem with studies of changed gene expression in *Giardia* trophozoites during interactions with intestinal epithelial cells *in vitro* are the control parasites; *Giardia* trophozoites do not do well in the growth mediums adapted for the intestinal epithelial cells. In this model, we pre-incubated the trophozoites in DMEM for 2 h prior to the addition to the Caco-2 cells to reduce the background effects on gene expression due to the change in medium. The levels of up- and down-regulation of gene expression is smaller in this study compared to what was earlier observed (Ringqvist et al., 2011), suggesting that a big part of the transcriptional changes in earlier studies can be attributed to the change of growth medium, but still 4-times more DEGs were identified in this study compared to the earlier studies (Emery-Corbin et al., 2020). Nevertheless, there are common transcriptional changes detected in all studies, even if different intestinal cell lines and methods for RNA analyses were used (Ringqvist et al., 2011; Ferella et al., 2014; Ma’ayeh et al., 2017). This makes it possible to summarize differential gene expression changes in *Giardia* trophozoites during host cell interactions and make some general comments. First, the largest changes in gene expression are induced by medium changes and not direct interaction with IECs. Second, the level of up- and down-regulation of RNA expression in *Giardia* is relatively small compared to other organisms, suggesting either a very tight level of regulation or post-transcriptional regulation. Third, up-regulation of metabolic, encystation and oxidative stress related genes is seen in all studies and this is most likely due to the stress induced during the interaction experiments. However, this type of stressors, oxidative and nutrient stress and induction of encystation, is also seen *in vivo* when mice are infected by *Giardia* WB trophozoites (Pham et al., 2017).

All earlier transcriptomal analyses of *Giardia*-host cell interactions have suggested an important role for proteases during *Giardia*-host interactions (Emery-Corbin et al., 2020). In this study, up-regulation was seen of a large number of cysteine proteases (CPs, encoded by ORFs 3169, 10217, 11209, 15564, 16160, 16779, 17516, 29304, 112831, 113303, 113656, 114165, 114773, 114915, and 137680). Most of these CPs have been shown to be up-regulated during host–parasite interactions *in vivo* (Pham et al., 2017) and to be more expressed in parasites recently axenized from human patients (4 generations) compared to parasites that have been grown for 50 generations *in vitro* (Ankarklev et al., 2015). Several recent studies have highlighted the importance of *Giardia* CPs in infections, specifically, the degradation of chemokines, antibodies, antimicrobial peptides, tight junction proteins and in encystation (Williams and Coombs, 1995; Jiménez et al., 2000; Coradi and Guimarães, 2006; Rodríguez-Fuentes et al., 2006; Carvalho et al., 2008; Ringqvist et al., 2011; Ma’ayeh and Brook-Carter, 2012; Ferella et al., 2014; Bhargava et al., 2015; Emery et al., 2016; Dubourg et al., 2018; Liu et al., 2018). It will be important to further characterize the CPs encoded by ORFs 10217, 16160, 16779, 17516, and 137680 in order to understand the virulence of *Giardia*.

The production of reactive oxygen species (ROS) represents one of the main epithelial cell defenses to fight off trophozoites early during infection (Ma’ayeh et al., 2015). Nevertheless, the ability of microaerophilic trophozoites to counteract host oxidative defenses has been investigated previously *in vitro*, highlighting the roles of the thioredoxin system, NADH oxidases, peroxiredoxins oxidoreductases, nitroreductases and nicotinamide co-factors in neutralizing host ROS (Ma’ayeh et al., 2015; Mastronicola et al., 2016; Pham et al., 2017). In fact, the genes encoding the above enzymes were differentially expressed at 1.5 h and thereafter (Supplementary Tables S2–S4), corroborating the above findings. Furthermore, the anti-oxidative stress responses have been also reported in trophozoites during mice infections, suggesting that *in vitro* results also can be seen *in vivo* (Pham et al., 2017).

Genes encoding hypothetical proteins have been found to be the main group of up-regulated genes in all gene expression studies of *Giardia*-host cell interactions (Ringqvist et al., 2011; Ma’ayeh and Brook-Carter, 2012; Pham et al., 2017) and also in this study hypothetical proteins dominated the DEGs. However, the hypothetical proteins are often *Giardia*-specific and have unknown function, which makes it difficult to make any conclusions about their function. A recent study modeled the structure of proteins encoded by 5,000 ORFs, including most hypothetical proteins, in *Giardia* using I-TASSER (Ansell et al., 2019). This changed the annotation of 212 hypothetical proteins and the new annotations have been used in this study. The large membrane protein encoded by ORF 114210 is glycosylated (Ratner et al., 2008) and the protein structure modeling using I-TASSER shows that it is similar to complement factor H (Ansell et al., 2019). Complement factor H is a large, soluble glycoprotein found in serum that suppresses reactions of the alternative pathway of the complement system to host cells (Parente et al., 2017). The complement system has been shown to be important in the immune defense against of *Giardia* in mice (Li et al., 2016; Coelho and Singer, 2018) and it is possible that this protein is used by the parasite to prevent complement-mediated immune mechanisms. Another gene, ORF5800, that earlier was annotated to encode a hypothetical protein, has been shown to be similar to lipid transporters in the StART family (Ansell et al., 2019). *Giardia* cannot produce most of its lipids and instead needs to scavenge them from the host (Jarroll et al., 1981; Mendez et al., 2015). Expression of genes involved in lipid metabolism in *Giardia* have been shown to be up-regulated when WB parasites infect mice (Pham et al., 2017) and this is also true for ORF5800. The role of the protein encoded by ORF5800 during *Giardia* infections will be interesting to study in more detail.

The *Giardia* gene family that shows the largest levels of differential gene expression during *Giardia*-host interactions *in vitro* and *in vivo* are the High-Cysteine Membrane Proteins (HCMPs). It is a yet uncharacterized family of proteins with high resemblance to the cysteine-rich Variant Surface Proteins (VSPs) (Davids et al., 2006). HCMPs often co-localize with VSPs in variable parts of the genome (Xu et al., 2020) and there are isolate-specific differences in the HCMP repertoires (Franzén et al., 2009; Ankarklev et al., 2015). Structurally, the VSPs have one conserved transmembrane domain, multiple extracellular CXXC motifs and a short cytoplasmic tail with the sequence CRGKA (Ankarklev et al., 2010). HCMPs also have multiple, extracellular CXXC motifs but often also multiple CXC motifs and the cytoplasmic domain is variable in size (Davids et al., 2006). Originally 61 HCMP genes were identified in the *Giardia* WB genome (Morrison et al., 2007) but in our new, more complete assembly of the WB genome we identified 116 HCMP genes (Xu et al., 2020). VSPs are involved in antigenic variation in order for the parasite to avoid being eliminated by the adaptive immune system and one main VSP is expressed on the cell surface of each trophozoite (Prucca and Lujan, 2009). The VSPs have been shown to be regulated epigenetically (Kulakova et al., 2006; Carranza et al., 2016) and post-transcriptionally by small RNAs (Prucca et al., 2008; Saraiya et al., 2014) but the understanding of VSP regulation is still limited. The HCMPs were first studied in the association with encystation (Davids et al., 2006) but we know now that they are also induced by other types of stressors (e.g., type of medium, temperature, drugs or oxidative stress) (Ansell et al., 2016; Einarsson et al., 2016b). One major limitation of earlier studies of HCMP gene expression has been the poor genome annotation but also limitations in the microarray, subtractive hybridization and serial-analyses of gene expression (SAGE) in differentiating HCMPs. Here we used the newly assembled WB genome (Xu et al., 2020), which is more complete and better annotated, in combination with RNAseq to show that 70 of the 116 HCMP genes in the WB isolate are differentially expressed during co-incubation with differentiated Caco-2 cells (Supplementary Table S5). In fact, certain HCMPs have earlier been shown to be up-regulated during interaction with other intestinal cell lines (HT29 and IEC-6) (Ferella et al., 2014; Ma’ayeh et al., 2017) and to be more highly expressed in the mouse intestine compared to growth *in vitro* in the standard medium TYDK (Pham et al., 2017) but this is the most complete study so far of HCMP gene expression. A summary of the data generated here together with earlier published data show that the HCMPs have different expression patterns in different conditions but we did not see any correlation between protein sequence similarity and expression profile (Supplementary Table S8). The expression patterns of the VSPs compared to the HCMPs suggest that the two groups display different types of regulation, even if there is some over-lap between the families (Supplementary Tables S2–S4). Only 27 of the 341 VSP genes in the *Giardia* WB genome (Xu et al., 2020) are up-regulated during co-incubation with differentiated Caco-2 cells and the level of up-regulation is lower than in the HCMP group (no VSPs among the 200 most up-regulated genes during the interaction compared to 13 HCMPs). Further studies are needed in order to define the relationship between VSPs and HCMPs and to see if there are sub-classes within each group with different expression profiles.

We used epitope-tagging to localize a few of the HCMPs which were up-regulated during interaction with IECs (e.g., ORFs 7715, 91707, and 115066). These HCMPs were plasma membrane-associated but they were also partially localized around the peripheral vesicles, ER and nuclear membrane. The localization HCMP91707 varied with generation numbers in culture, which suggests that certain HCMPs can display a dynamic localization between internal and external membranes. Some HCMPs are also released into the surrounding environment during host cell interactions (Ma’ayeh et al., 2017). The presence of epidermal growth factor (EGF)-like domains within many of the HCMPs might relate to possible functions associated with this dynamic localization inside and outside the giardial cell. EGF-like domains are usually present in secreted proteins but they can function intracellularly upon ligand binding to mediate the release of transcriptional factors or modulate transcription within the nucleus (e.g., NOTCH signaling) (Kiyota and Kinoshita, 2004; Lai, 2004). This domain is also associated with diverse functions including calcium binding (Handford et al., 1991), adhesion (Stenflo, 1991), protein–protein interactions and intracellular and extracellular signaling (Wouters et al., 2005). It will be interesting to identify which exact role HCMPs and EGF-like domain have in *Giardia*.

This and earlier studies (Sonda et al., 2010) suggest a putative role of NAD^+^-independent HDACs in the regulation of certain HCMPs, implying that HCMPs are regulated at chromatin structure level, similar to VSPs. HCMPs are structurally similar to VSP and they have a proximal chromosomal location to VSPs throughout the genome (Adam, 2000; Morrison et al., 2007). Interestingly, a unique NAD^+^-independent histone deacetylase has been found to modulate VSP switching (Carranza et al., 2016). Blocking this HDAC with a specific drug induced the expression of encystation-specific genes, VSPs and certain HCMPs (Sonda et al., 2010). Epigenetic regulation is a very complicated process and in-depth analysis of histone modifications, DNA methylation and chromatin-modifying enzymes is warranted to draw a comprehensive conclusion on HCMP regulation during different stages of the parasite life-cycle and during infection.

Iron is an essential trace-metal for all organisms and it is a well-known regulator of virulence genes in many pathogenic bacteria (Begg, 2019) but also in pathogenic protozoa, including *Entamoeba histolytica*, *Trichomonas vaginalis*, *Trypanosoma*, and *Leishmania* (Niu et al., 2016; Gastelum-Martínez et al., 2018; Paiva et al., 2018). This is because iron is an essential constituent of many proteins in the pathogens, including metabolic and antioxidant enzymes and virulence factors (Arroyo et al., 2015). In the duodenum, were ferric iron (Fe^2+^) absorption occurs in humans, the level of accessible iron is limited for invading pathogens, resulting in a competition for free iron with the host cells, and iron has been shown to be a major regulator of virulence genes in intestinal bacteria (Begg, 2019). Here, we studied the effect of reducing the iron concentration in the growth medium or its fixation by a chelation agent on *Giardia* trophozoites. The level of total iron in the standard *Giardia* growth medium TYDK is very high (5 mg/ml) but the levels of iron available for *Giardia in vivo* is much lower (Deschemin et al., 2016). Thus, it will be important to determine what *Giardia* genes that are differentially expressed at lower levels of free-iron compared to in the standard TYDK medium. In the first data set we did not add extra iron to the *Giardia* growth medium and this reduced the total levels of iron threefold. This resulted in 225 DEGs, including up-regulation of several genes that earlier have been suggested to be virulence genes (Supplementary Table S6). The level of up- and down-regulation of RNA expression in *Giardia* is relatively small compared to other organisms as noted above and this can also be seen in the analysis of iron-regulated genes. We detected 613 DEGs in the chelator treated parasites compared to cells grown in TYDK but the effect was mainly a down-regulation of a broad repertoire of genes. The addition of a metal ion chelator in to the medium should reduce the free iron but it is also binding other metal ions, increasing the risk of non-iron related responses. This calls for alternative methods for specific reduction of iron to lower levels than in the standard TYDK medium. We noticed the induction of many HCMPs and VSPs in both treatments, indicating that HCMP and VSP genes transcription can also be regulated by iron. It will be interesting to see whether there are iron response elements within the sequences of these induced genes similar to that in *Entamoeba* (Soto-Castro et al., 2017). Furthermore, the withdrawal of iron resulted in the enrichment of functional groups relating to iron functions, such as the oxidoreductase activity, ATPase activity, nucleoside triphosphatase activity, and pyrophosphatase activity and nucleoside metabolism. It was interesting to see the pyrophosphatase activity group enriched since the use of pyrophosphate in *Giardia* produces more ATP in energy limiting conditions (Ma’ayeh and Brook-Carter, 2012). This overall indicate a compensatory response for energy, antioxidant functions and the acquisition of nucleosides to counteract iron withdrawal. The role of iron during *Giardia* infections is not well-characterized but our expression datasets can be start for further studies of this important factor.

To conclude, gene expression analyses of *Giardia* trophozoites during interaction with IECs *in vitro* have identified a group of genes that are up-regulated during interactions. This group contains genes encoding cysteine proteinases, oxidative stress response proteins, metabolic proteins and certain HCMP genes. We have also identified genes that are regulated by the level of iron in the growth medium. The recent development of CRISPR repression and mutagenesis systems (Lin et al., 2019; McInally et al., 2019) will make it possible to define the role of these genes in *Giardia’s* virulence. Mutant parasites can be tested *in vitro* in enteroid systems or *in vivo* in mice or gerbils and this can lead to a more detailed molecular understanding of giardiasis.

## Data Availability Statement

The data sets generated here can be found at Gene Expression Omnibus (GEO); accession ID GSE144004 for the interaction experiment and GSE136820 for the iron depletion experiment.

## Author Contributions

DP, SM, FX, MF, SC, and JL performed the experiments, analyzed the data, and wrote parts of the manuscript. SS conceived and designed the experiments and wrote the first draft of the manuscript. All authors revised the manuscript.

## Conflict of Interest

The authors declare that the research was conducted in the absence of any commercial or financial relationships that could be construed as a potential conflict of interest.

## Footnotes

1 http://support.illumina.com/sequencing/sequencing_software/bcl2fastq-conversion-software.html
