# Supplementary material for: High Cysteine Membrane Proteins (HCMPs) Are Up-Regulated During Giardia-Host Cell Interactions
Source: Front Genet. 2020 Aug 18;11:913. doi: 10.3389/fgene.2020.00913 (PMC7461913; doi:10.3389/fgene.2020.00913)
Supplement: Supplementary file 9 [file Image_1.pdf]

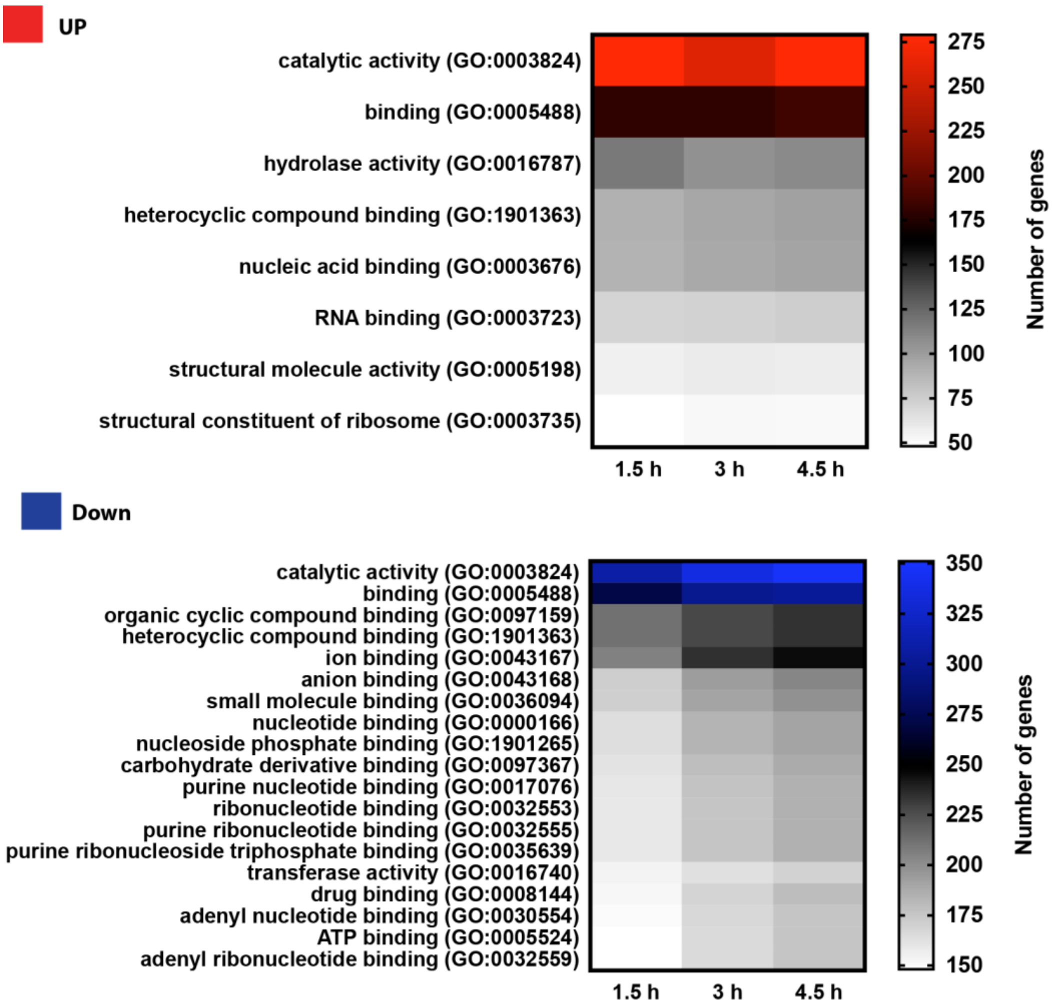

Figure S1. Gene Ontology (GO) analysis of DEGs in *Giardia intestinalis* for the three time points of interaction represented in the form of a heat map. Up-regulated genes are shown with in red while down-regulated genes are blue. Both maps (Up- and Down-regulated) show significant enrichment for molecular functions.
