# Supplementary material for: High Cysteine Membrane Proteins (HCMPs) Are Up-Regulated During Giardia-Host Cell Interactions
Source: Front Genet. 2020 Aug 18;11:913. doi: 10.3389/fgene.2020.00913 (PMC7461913; doi:10.3389/fgene.2020.00913)
Supplement: Supplementary file 10 [file Image_2.pdf]

A

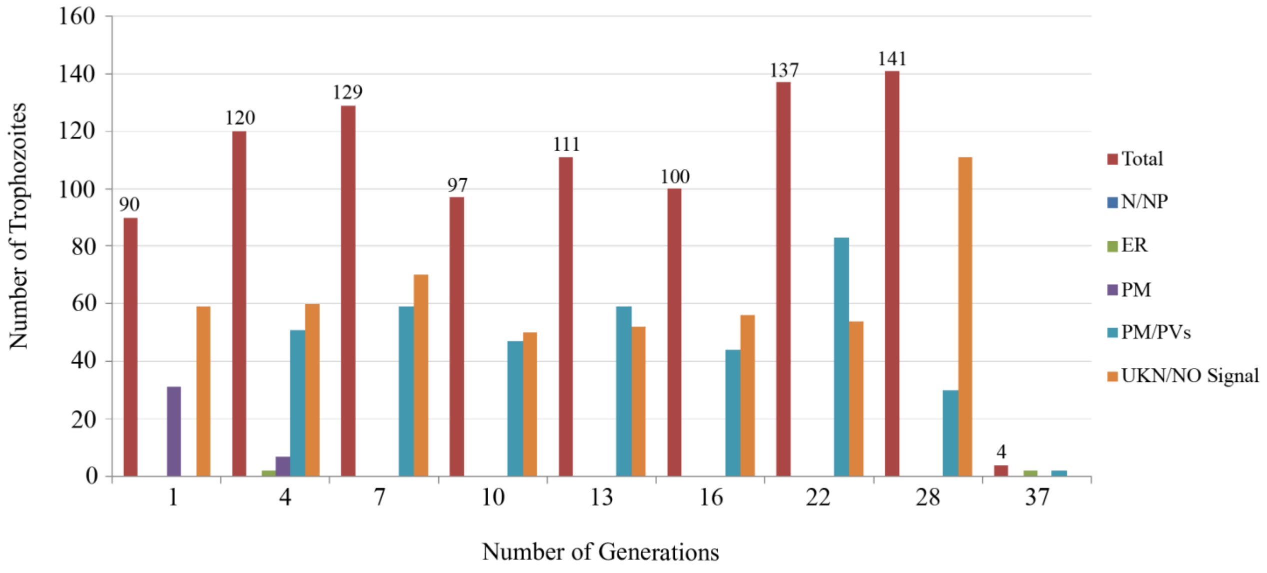

B

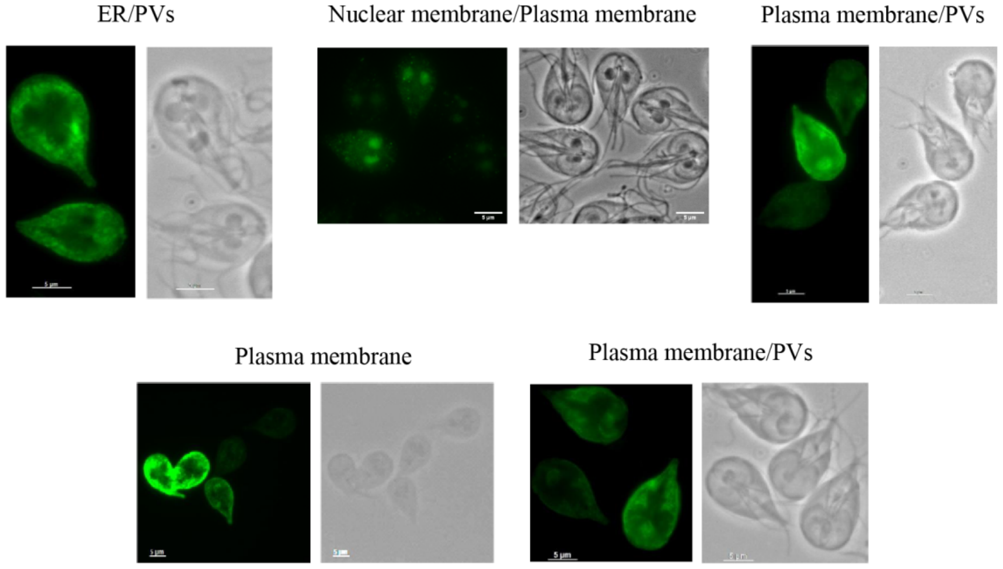

Figure S2. *Giardia* trophozoites were transfected with a plasmid vector over-expressing the C-terminal HA-tagged HCMP 91707 under selection of puromycin. Two hundred cells were counted each time on a fluorescent microscope for 37 generations and the localization was determined. N/NP:Nucleus/Nuclear periphery, ER:Endoplasmic reticulum, PM:Plasma Membrane, PM/PVs:Plasma Membrane/Peripheral Vacuoles, UKN/No:Uknown Signal/No Signal
