# Supplementary figures and images for: High Cysteine Membrane Proteins (HCMPs) Are Up-Regulated During Giardia-Host Cell Interactions
Source: Front Genet. 2020 Aug 18;11:913. doi: 10.3389/fgene.2020.00913 (PMC7461913; doi:10.3389/fgene.2020.00913)

**7715-Int**

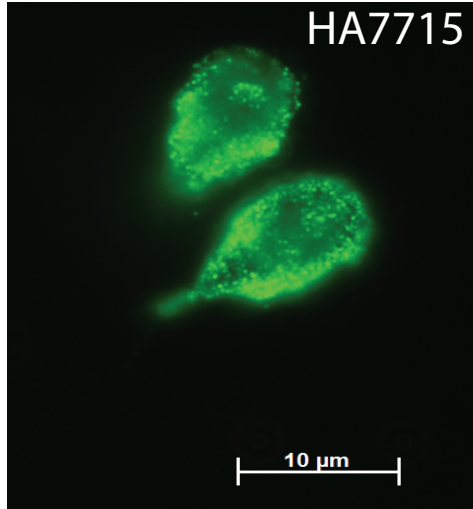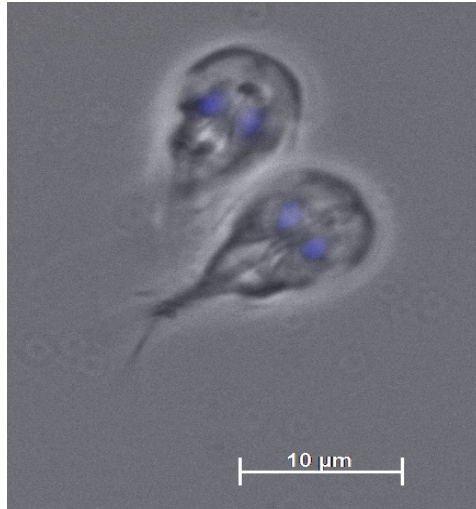

**91707-Int**

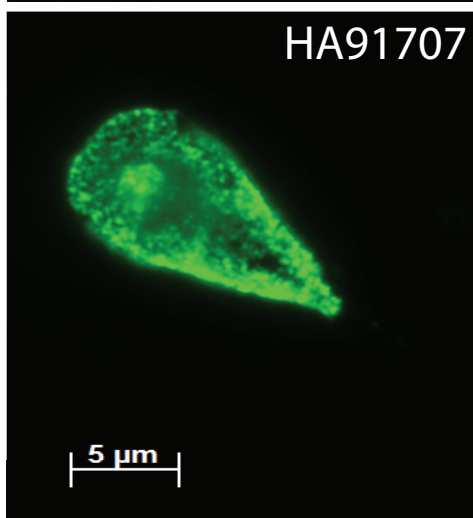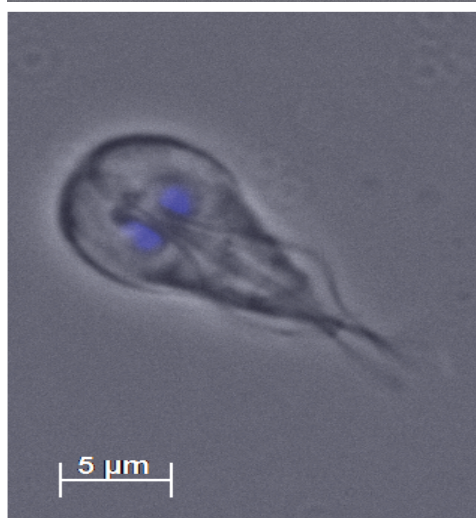

**115066-Int**

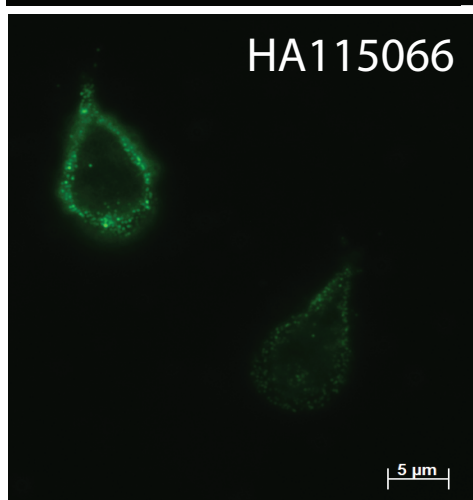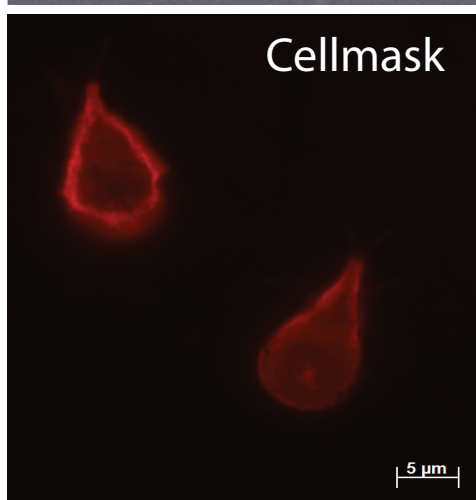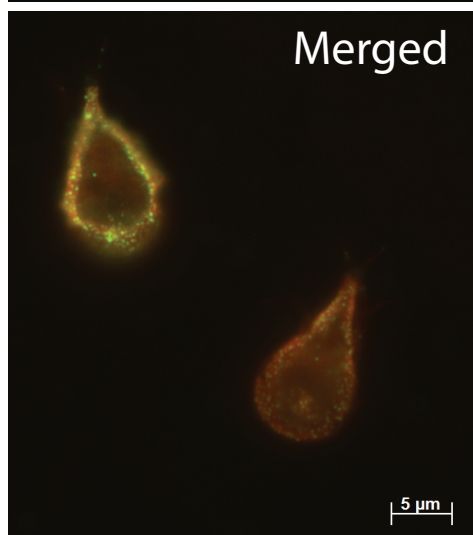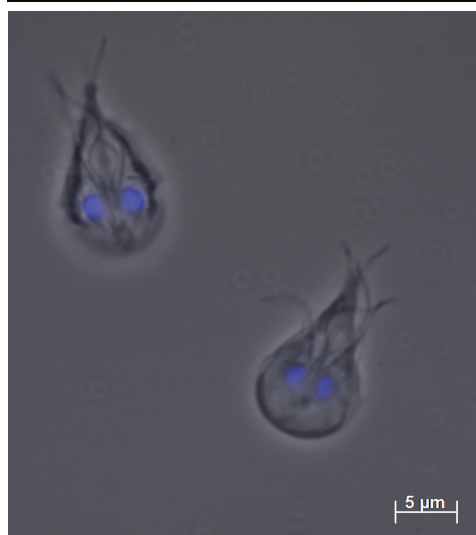

**Figure S3.** Localization of integrated, C-terminally tagged HCMPs.

Supplement: Supplementary file 11 [file Image_3.pdf]
